# Supplementary material for: Healthcare workers’ experiences with COVID-19-related prevention and control measures in Tanzania
Source: PLOS Glob Public Health. 2023 Dec 5;3(12):e0002678. doi: 10.1371/journal.pgph.0002678 (PMC10697532; doi:10.1371/journal.pgph.0002678)
Supplement: S1 Appendix — Table A: Odds ratios for Likert style questions to be Agree/Strongly Agree Table B: Odds ratios for more Likert style questions to be Agree/Strongly Agree Table C: Reported Challenges from Healthcare workers Table D: Health care worker questionnaire. (DOCX) [file pgph.0002678.s002.docx]

**Table A: Health care worker questionnaire.**

| **Provider Background Questions: We would like to ask several questions about your background and the health facility designation where you are PRIMARILY engaged** | | | |
| --- | --- | --- | --- |
| B01 | What is your gender? | 0 | Male |
|  |  | 1 | Female |
| B02 | What is your Age? |  | |
| B03 | What year did you finish your studies and begin providing care? |  | |
| B04 | What is your cadre of healthcare provider | 1 | Nurse |
|  |  | 2 | Clinical Officer |
|  |  | 3 | Medical Officer |
|  |  | 4 | Technician |
|  |  | 5 | Pharmacist |
|  |  | 6 | Other |
| B05 | Please specify your cadre |  | |
| B06 | Does the healthcare facility serve mostly rural or urban patients | 0 | Rural |
|  |  | 1 | Urban |
| B07 | Which best describes the health facility | 1 | Dispensary |
|  |  | 2 | Health Center |
|  |  | 3 | Hospital |
|  |  | 4 | Referral Hospital |
|  |  | 5 | Private Clinic |
| B08 | Is the facility run by the government, an NGO or religious organization, or private | 1 | Government |
|  |  | 2 | NGO or religious |
|  |  | 3 | Private |
| B09 | Regional |  |  |
| B10 | District |  |  |
| B11 | Is the health facility designated to offer COVID-19 Services | 0 | No  [survey continues with FR01] |
|  |  | 1 | Yes  [survey continues with PB 10] |
| B12 | Since your facility was designated to offer COVID-19 Services, has it also offered non-COVID-19 services? | 0 | No |
|  |  | 1 | Yes |

| **Please answer the following to the best of your knowledge regarding the health facility where you do most of your work:** | | | | |
| --- | --- | --- | --- | --- |
| C01 | Is the facility where you work approved/authorized to carry out COVID-19 tests and present results | 0 | No | |
|  |  | 1 | Yes | |
| C02 | To your knowledge, have there been any cases of COVID-19 treated at your health facility? | 0 | No  [survey continues with FR03] | |
|  |  | 1 | Yes  [survey continues with FR02] | |
| C03 | Are these cases suspected, Confirmed, or both | 1 | Suspected | |
|  |  | 2 | Confirmed | |
|  |  | 3 | There are both suspected and confirmed cases at the health facility | |
| C04a | To your knowledge has your health facility received any directives from the government regarding guidelines for treating or controlling the spread of COVID-19 | 0 | No  [survey continues with FR07] | |
|  |  | 1 | Yes  [survey continues with FR04] | |
| C04b | From which level of government did the guidelines originate? Select all that are relevant | □ | Ministry of Health (National) | |
|  |  | □ | Regional Authority | |
|  |  | □ | District Authority | |
|  |  | □ | Local (municipal or Village level authority) | |
|  |  | □ | Other: (Please Enter) | |
| C04c | From which level of government (specify) |  |  | |
| C04d | Was this guidance general or specific to programs? | 0 | General | |
|  |  | 1 | AIDS Control Program | |
|  |  | 2 | Other specific Program (please add) | |
| C04e | Please specify, what were these special program |  | | |
| C04f | Which guidelines did this communicate? Please select all that you are aware of being communicated regardless of implementation at your facility | General | | |
|  |  | □ | PPE use training | |
|  |  | □ | PPE availability | |
|  |  | □ | Management of potentially contaminated waste | |
|  |  | □ | Masking of health care workers and clients with respiratory symptoms | |
|  |  | All patient care changes | | |
|  |  | □ | Optimizing care and treatment clinic (CTC) space to reduce close contact among people including rearranging waiting and triage areas to ensure physical distancing between clients | |
|  |  | □ | Prioritization of care for patients with comorbidities | |
|  |  | □ | Implement block system appointments or enhance clinical appointments arrangements to reduce crowding | |
|  |  | □ | Use telephone/mobile or virtual visits for routine or non-urgent care, adherence counselling, and reporting of medication side effects | |
|  |  | □ | Postpone routine medical and laboratory visits as much as possible | |
|  |  | □ | Enhance or scale-up multi-month prescription and dispensing for chronic care of stable clients, especially for patients on ART | |
|  |  | COVID-19 Treatment or referral | | |
|  |  | □ | COVID-19 specific Infection Prevention and Control (ICP) | |
|  |  | □ | Definitions of suspected COVID-19 cases | |
|  |  | □ | COVID-19 Triage | |
|  |  | □ | Collection of specimens for laboratory diagnosis | |
|  |  | □ | COVID-19 Treatment protocol for early supportive therapy and monitoring | |
|  |  | □ | Syndromic treatment and severe case management | |
|  |  | □ | Referral pathways for all COVID-19 cases if not treated at the facility | |
|  |  | □ | Referral pathways for severe COVID-19 cases if severity requires more care | |
|  |  | □ | Discharge criteria | |
|  |  | □ | Other(s): Please list | |
| C05 | For now, we will like to understand the implementation of the guidelines at your health facility. Please select the guidelines for treatment and prevention of COVID-19 implemented at your health facility. These could be the guidelines from the government or guideline specific for this health facility |  |  |  |
| C05a | Others (please specify) |  |  |  |
| C08 | [If respondents selected “Temporary Suspension of services” in FR07 they are supplied with this follow up question, otherwise the survey will skip to FR09]  Please select all services that have been suspended and the date that it went into effect. Please enter approximate date if you are unsure of the exact date. | □ | Testing for chronic conditions like hypertension or diabetes | Date |
|  |  | □ | Cancer treatments | Date |
|  |  | □ | Ophthalmology | Date |
|  |  | □ | Non-Emergency Surgery | Date |
|  |  | □ |  | Date |
|  |  | □ | Other: (please specify) | Date |
| C08a | Others (specify) |  |  | |
| C09 | Has the health facility seen a change in patient volume? | 0 | No, patient volume is about the same | |
|  |  | 1 | Yes, patient volume has INCREASED | |
|  |  | 2 | Yes, patient volume has DECREASED | |
| C10 | Is there COVID-19 Testing capacity at the facility? | 0 | No | |
|  |  | 1 | Yes | |
| C11 | To your knowledge, are patients who are presenting with possible COVID-19 symptoms turned away from care or advised to seek care elsewhere from someone at the facility? | 0 | No  [survey continues at FR13] | |
|  |  | 1 | Yes  [survey continues at FR12] | |
| C12 | Are suspected COVID-19 patients referred to somewhere specific? | 0 | No | |
|  |  | 1 | Yes | |
| C13 | Is there a nearby facility that is designated as the COVID-19 treatment centre? | 0 | No | |
|  |  | 1 | Yes | |
| C14 | What PPE is available for HCW at your facility? Please check all that are available | □ | Medical/Surgical mask | |
|  |  | □ | Gloves | |
|  |  | □ | long-sleeved gown | |
|  |  | □ | Fit Tested N95 mask or equivalent respirators | |
|  |  | □ | Eye Protection | |
|  |  | □ | Medical scrubs to change immediately after shifts | |
| C15 | Have you experienced any violence or threats towards healthcare workers? | 0 | No | |
|  |  | 1 | Yes | |
| C16 | Some HCW are experiencing additional challenges, please select any that you are | □ | stress from working with an infectious disease including the polices used to control spread | |
|  |  | □ | exhausted from wearing protective gear | |
|  |  | □ | fatigue from workload increases as critically ill patients require substantial care | |
|  |  | □ | concern or uncertainty of getting infected or infecting others | |
|  |  | □ | emotional distress from powerlessness if patients worsen or die despite efforts | |
|  |  | □ | emotional distress from high levels of mortality | |
| C17 | Have you experienced any other challenges? | [open ended, room for a few line response] | | |
| C18 | Is there anything else you want to share with us? | [open ended, room for a few line response] | | |

| **We now present a series of statements and ask you to express your agreement on a scale from Strongly agree, to strongly disagree. This is your own personal opinion, and will be kept strictly anonymized so please be completely honest.** | | | | | | |
| --- | --- | --- | --- | --- | --- | --- |
| D01 | The policies in my place of work are adequate to provide protection for HCW | strongly disagree | disagree | neutral | agree | strongly agree |
| D02 | The policies in my place of work are adequate to provide protection for patients and clients | strongly disagree | disagree | neutral | agree | strongly agree |
| D03 | We have adequate stock of PPE | strongly disagree | disagree | neutral | agree | strongly agree |
| D04 | I feel as safe at work as usual | strongly disagree | disagree | neutral | agree | strongly agree |
| D05 | I am afraid or concerned about violence or threats towards healthcare workers | strongly disagree | disagree | neutral | agree | strongly agree |
| D06 | The health facility is adequately prepared to treat COVID-19 patients | strongly disagree | disagree | neutral | agree | strongly agree |
| D07 | The directions we are given regarding COVID-19 are clear | strongly disagree | disagree | neutral | agree | strongly agree |
| D08 | I know who I can ask about COVID-19 policies if I have more questions | strongly disagree | disagree | neutral | agree | strongly agree |
| D09 | I do not feel that my concerns regarding COVID-19 are being addressed | strongly disagree | disagree | neutral | agree | strongly agree |
| D10 | I am concerned about putting my family at risk because of my work | strongly disagree | disagree | neutral | agree | strongly agree |
| D11 | I am experiencing an increased amount of stress due to working with COVID | strongly disagree | disagree | neutral | agree | strongly agree |
| D12 | I feel it is my duty to help patients even at the risk to myself | strongly disagree | disagree | neutral | agree | strongly agree |
| D13 | My facility management is concerned about my emotional well-being | strongly disagree | disagree | neutral | agree | strongly agree |

**Table B: Reported Challenges from Healthcare workers**

|  | Total  N (%) | Dispensary  N (%) | Health Center  N (%) | Hospital  N (%) | Referral Hospital  N (%) | Clinics  N (%) | P-Value |
| --- | --- | --- | --- | --- | --- | --- | --- |
| **Experienced Threats towards HCW** | 1085 (15.8) | 258 (11.9) | 312 (16.9) | 252 (16.1) | 208 (21.2) | 55 (16.7) | <0.000 |
| **Increased Stress from working with infectious disease** | 5023 (73.0) | 1525 (70.6) | 1363 (73.6) | 1186 (75.9) | 725 (74.0) | 224 (67.9) | 0.001 |
| **Physically exhausted from wearing protective gear** | 1534 (22.3) | 459 (21.3) | 383 (20.7) | 392 (25.1) | 221 (22.6) | 79 (23.9) | <0.000 |
| **Fatigue From Workload Increase** | 1043 (15.2) | 236 (10.9) | 245 (13.2) | 306 (19.6) | 202 (20.6) | 54 (16.4) | <0.000 |
| **Concern about getting infected or infected others** | 4411 (64.1) | 1253 (58.0) | 1222 (66.0) | 1089 (69.7) | 661 (67.5) | 186 (56.4) | <0.000 |
| **Emotional Distress from powerlessness if patients deteriorate** | 2207 (32.1) | 535 (24.8) | 549 (29.7) | 626 (40.1) | 400 (40.8) | 97 (29.4) | <0.000 |
| **Emotional Distress from high levels of mortality** | 1558 (22.6) | 405 (18.8) | 398 (21.5) | 416 (26.6) | 261 (26.6) | 78 (23.6) | <0.000 |

**Table C: Odds ratios for Likert style questions to be Agree/Strongly Agree**

|  | Adequate. Policies for HCW | Adequate. Policies for HCW | Adequate  PPE | Feel as safe as usual | (Flipped) concerned about violence | Adequately  Prepared for COVID patients | COVID directions clear |
| --- | --- | --- | --- | --- | --- | --- | --- |
| Female | 0.873 | 0.982 | 1.041 | 0.969 | 0.990 | 1.136 | 1.256^*^ |
|  | (0.0663) | (0.0483) | (0.0918) | (0.0751) | (0.0522) | (0.0781) | (0.142) |
| Age |  |  |  |  |  |  |  |
| 18-29 | Ref | Ref | Ref | Ref | Ref | Ref | Ref |
| 30-49 | 0.751^***^ | 0.764^***^ | 0.891 | 0.769^***^ | 1.152^**^ | 0.910 | 0.753^***^ |
|  | (0.0511) | (0.0376) | (0.0585) | (0.0548) | (0.0555) | (0.0529) | (0.0644) |
| 50+ | 0.814 | 0.865 | 1.074 | 0.957 | 1.375^*^ | 1.022 | 0.974 |
|  | (0.123) | (0.140) | (0.183) | (0.146) | (0.211) | (0.124) | (0.223) |
| Cadre |  |  |  |  |  |  |  |
| Nurse | Ref | Ref | Ref | Ref | Ref | Ref | Ref |
| Clinical Officer | 0.942 | 1.006 | 0.826^*^ | 0.875^*^ | 0.914 | 0.885 | 1.022 |
|  | (0.0631) | (0.0636) | (0.0675) | (0.0574) | (0.0706) | (0.0646) | (0.106) |
| Medical Officer | 0.810^**^ | 0.824^**^ | 0.594^***^ | 0.611^***^ | 0.742^**^ | 0.666^***^ | 0.851 |
|  | (0.0578) | (0.0485) | (0.0439) | (0.0425) | (0.0808) | (0.0482) | (0.115) |
| Technician | 1.535^***^ | 1.602^***^ | 1.144 | 1.349^**^ | 1.094 | 1.185 | 1.362 |
|  | (0.154) | (0.143) | (0.125) | (0.138) | (0.125) | (0.120) | (0.247) |
| Pharmacist | 1.483^**^ | 1.534^**^ | 1.153 | 1.571^**^ | 0.963 | 1.293 | 1.094 |
|  | (0.197) | (0.232) | (0.141) | (0.223) | (0.128) | (0.170) | (0.273) |
| Other | 1.538^***^ | 1.580^***^ | 0.795^*^ | 1.454^***^ | 0.945 | 0.842 | 1.302 |
|  | (0.170) | (0.156) | (0.0804) | (0.155) | (0.0884) | (0.0886) | (0.247) |
| Urban | 1.112 | 1.075 | 1.107 | 0.990 | 0.859^**^ | 1.146 | 1.081 |
|  | (0.0997) | (0.107) | (0.120) | (0.0933) | (0.0464) | (0.0965) | (0.0764) |
| Health Facility Level |  |  |  |  |  |  |  |
| Dispensary | Ref | Ref | Ref | Ref | Ref | Ref | Ref |
| Health Centre | 0.853^*^ | 0.844^*^ | 0.816^**^ | 0.789^***^ | 0.970 | 1.107 | 0.754^*^ |
|  | (0.0676) | (0.0621) | (0.0627) | (0.0407) | (0.0609) | (0.0894) | (0.0875) |
| Hospital | 0.830 | 0.800^*^ | 0.744^*^ | 0.738^**^ | 1.046 | 1.304^*^ | 0.880 |
|  | (0.0922) | (0.0775) | (0.0919) | (0.0723) | (0.109) | (0.158) | (0.101) |
| Referral Hospital | 0.816 | 0.789 | 0.924 | 0.710^*^ | 0.994 | 1.500 | 0.779 |
|  | (0.137) | (0.119) | (0.195) | (0.100) | (0.121) | (0.373) | (0.130) |
| Private Clinic | 0.971 | 0.972 | 1.092 | 1.108 | 0.674^**^ | 1.136 | 0.878 |
|  | (0.157) | (0.131) | (0.140) | (0.174) | (0.0928) | (0.168) | (0.188) |
| Facility Ownership |  |  |  |  |  |  |  |
| Government | Ref | Ref | Ref | Ref | Ref | Ref | Ref |
| NGO or religious | 1.415^**^ | 1.405^**^ | 1.544^***^ | 1.454^***^ | 1.179 | 1.535^***^ | 1.115 |
|  | (0.165) | (0.173) | (0.150) | (0.143) | (0.124) | (0.165) | (0.141) |
| Private | 1.482^***^ | 1.608^***^ | 1.662^***^ | 1.443^***^ | 1.112 | 1.453^***^ | 1.136 |
|  | (0.111) | (0.155) | (0.0888) | (0.0968) | (0.0972) | (0.145) | (0.160) |
| Designated for COVID Care Services | 1.326^***^ | 1.376^***^ | 1.006 | 1.298^***^ | 1.287^**^ | 1.845^***^ | 1.629^***^ |
|  | (0.0888) | (0.101) | (0.0734) | (0.0786) | (0.113) | (0.137) | (0.150) |
| COVID-19 Cases |  |  |  |  |  |  |  |
| No Cases  Suspected | Ref | Ref | Ref | Ref | Ref | Ref | Ref |
|  | 0.924 | 0.976 | 0.886^*^ | 0.903 | 0.854^*^ | 1.311^***^ | 0.845^*^ |
| Some or All Cases Confirmed | (0.0615) | (0.0809) | (0.0516) | (0.0579) | (0.0609) | (0.0843) | (0.0724) |
|  | 0.984 | 1.107 | 1.060 | 0.981 | 0.998 | 1.911^***^ | 0.932 |
|  | (0.0796) | (0.0900) | (0.0812) | (0.0708) | (0.0665) | (0.116) | (0.104) |
|  |  |  |  |  |  |  |  |
| Guidelines for COVID-19 |  |  |  |  |  |  |  |
| None | Ref | Ref | Ref | Ref | Ref | Ref | Ref |
| Ministry of Health (National) | 1.178^*^ | 1.054 | 1.136^*^ | 1.217^**^ | 1.094 | 1.091 | 1.593^***^ |
|  | (0.0848) | (0.0755) | (0.0723) | (0.0765) | (0.0693) | (0.0854) | (0.168) |
| Regional | 0.920 | 0.931 | 0.925 | 0.916 | 0.883 | 0.956 | 0.943 |
|  | (0.0908) | (0.0807) | (0.0724) | (0.0748) | (0.0581) | (0.0779) | (0.133) |
| District | 0.955 | 0.904 | 0.898 | 0.901 | 1.185^*^ | 0.944 | 1.074 |
|  | (0.0736) | (0.0649) | (0.0582) | (0.0672) | (0.0848) | (0.0673) | (0.110) |
| Municipal/Village | 1.219 | 1.157 | 0.894 | 1.444^***^ | 1.049 | 1.173^*^ | 1.240 |
|  | (0.130) | (0.135) | (0.0882) | (0.157) | (0.0954) | (0.0882) | (0.199) |
| Observations | 6878 | 6878 | 6878 | 6878 | 6878 | 6878 | 6878 |

Exponentiated coefficients; Standard errors in parentheses

^*^ *p* < 0.05, ^**^ *p* < 0.01, ^***^ *p* < 0.001

**Table D: Odds ratios for more Likert style questions to be Agree/Strongly Agree**

|  | Know who to ask Re: COVID Policies | (Flipped)  Concerns Re: COVID are not  addressed | (Flipped) Concerned putting family at risk | (Flipped) experiencing increased Stress | My duty is to help my patients even if risk | Management Concerned for emotional well-being |
| --- | --- | --- | --- | --- | --- | --- |
| Female | 1.033 | 1.244^***^ | 1.028 | 1.199^***^ | 1.136 | 1.136 |
|  | (0.0836) | (0.0659) | (0.0544) | (0.0552) | (0.0781) | (0.0781) |
| Age |  |  |  |  |  |  |
| 18-29 | Ref | Ref | Ref | Ref | Ref | Ref |
| 30-49 | 0.942 | 0.903 | 0.797^***^ | 0.953 | 0.910 | 0.910 |
|  | (0.0742) | (0.0613) | (0.0440) | (0.0502) | (0.0529) | (0.0529) |
| 50+ | 1.233 | 0.947 | 0.899 | 0.861 | 1.022 | 1.022 |
|  | (0.261) | (0.152) | (0.148) | (0.126) | (0.124) | (0.124) |
| Cadre |  |  |  |  |  |  |
| Nurse | Ref | Ref | Ref | Ref | Ref | Ref |
| Clinical Officer | 0.987 | 1.100 | 0.849^*^ | 0.936 | 0.885 | 0.885 |
|  | (0.122) | (0.0626) | (0.0569) | (0.0785) | (0.0646) | (0.0646) |
| Medical Officer | 0.869 | 0.680^***^ | 0.503^***^ | 0.743^***^ | 0.666^***^ | 0.666^***^ |
|  | (0.113) | (0.0528) | (0.0445) | (0.0606) | (0.0482) | (0.0482) |
| Technician | 1.267 | 1.149 | 1.354^***^ | 1.251^*^ | 1.185 | 1.185 |
|  | (0.176) | (0.136) | (0.108) | (0.127) | (0.120) | (0.120) |
| Pharmacist | 0.789 | 1.027 | 1.221 | 1.039 | 1.293 | 1.293 |
|  | (0.137) | (0.107) | (0.140) | (0.109) | (0.170) | (0.170) |
| Other | 1.394^*^ | 1.200^*^ | 1.351^**^ | 0.854 | 0.842 | 0.842 |
|  | (0.231) | (0.106) | (0.151) | (0.0923) | (0.0886) | (0.0886) |
| Urban | 0.872 | 1.005 | 0.864^*^ | 0.925 | 1.146 | 1.146 |
|  | (0.0873) | (0.0629) | (0.0587) | (0.0743) | (0.0965) | (0.0965) |
| Health Facility Level |  |  |  |  |  |  |
| Dispensary | Ref | Ref | Ref | Ref | Ref | Ref |
| Health Centre | 0.739^**^ | 0.865 | 0.845^*^ | 1.063 | 1.107 | 1.107 |
|  | (0.0716) | (0.0660) | (0.0565) | (0.0809) | (0.0894) | (0.0894) |
| Hospital | 0.709^**^ | 1.039 | 0.783^**^ | 1.180 | 1.304^*^ | 1.304^*^ |
|  | (0.0811) | (0.0879) | (0.0699) | (0.106) | (0.158) | (0.158) |
| Referral Hospital | 0.609^*^ | 0.974 | 0.639^*^ | 0.960 | 1.500 | 1.500 |
|  | (0.133) | (0.112) | (0.118) | (0.173) | (0.373) | (0.373) |
| Private Clinic | 0.871 | 1.098 | 1.034 | 1.055 | 1.136 | 1.136 |
|  | (0.217) | (0.123) | (0.169) | (0.152) | (0.168) | (0.168) |
| Facility Ownership |  |  |  |  |  |  |
| Government | Ref | Ref | Ref | Ref | Ref | Ref |
| NGO or religious | 0.918 | 1.048 | 1.003 | 1.011 | 1.535^***^ | 1.535^***^ |
|  | (0.112) | (0.0984) | (0.0990) | (0.0937) | (0.165) | (0.165) |
| Private | 1.014 | 1.084 | 1.010 | 1.055 | 1.453^***^ | 1.453^***^ |
|  | (0.137) | (0.0856) | (0.108) | (0.0835) | (0.145) | (0.145) |
| Designated for COVID Care Services | 1.632^***^ | 1.478^***^ | 1.341^***^ | 1.060 | 1.845^***^ | 1.845^***^ |
|  | (0.136) | (0.0987) | (0.0869) | (0.0751) | (0.137) | (0.137) |
| COVID-19 Cases |  |  |  |  |  |  |
| No Cases | Ref | Ref | Ref | Ref | Ref | Ref |
| Suspected | 0.924 | 0.752^***^ | 0.887 | 0.723^***^ | 1.311^***^ | 1.311^***^ |
|  | (0.0850) | (0.0463) | (0.0600) | (0.0622) | (0.0843) | (0.0843) |
| Some or All Cases Confirmed | 1.143 | 0.781^***^ | 0.901 | 0.609^***^ | 1.911^***^ | 1.911^***^ |
|  | (0.116) | (0.0520) | (0.0757) | (0.0519) | (0.116) | (0.116) |
| Guidelines for COVID-19 |  |  |  |  |  |  |
| None | Ref | Ref | Ref | Ref | Ref | Ref |
| Ministry of Health (National) | 1.825^***^ | 1.094 | 1.055 | 0.966 | 1.091 | 1.091 |
|  | (0.198) | (0.0843) | (0.0563) | (0.0648) | (0.0854) | (0.0854) |
| Regional | 1.139 | 0.917 | 0.806^**^ | 0.837 | 0.956 | 0.956 |
|  | (0.115) | (0.0664) | (0.0609) | (0.0811) | (0.0779) | (0.0779) |
| District | 1.261^*^ | 1.032 | 0.918 | 0.933 | 0.944 | 0.944 |
|  | (0.130) | (0.0845) | (0.0619) | (0.0784) | (0.0673) | (0.0673) |
| Municipal/Village | 1.642^***^ | 1.153 | 1.072 | 1.079 | 1.173^*^ | 1.173^*^ |
|  | (0.241) | (0.132) | (0.127) | (0.110) | (0.0882) | (0.0882) |
|  |  |  |  |  |  |  |
| Observations | 6878 | 6878 | 6878 | 6878 | 6878 | 6878 |

Exponentiated coefficients; Standard errors in parentheses

^*^ *p* < 0.05, ^**^ *p* < 0.01, ^***^ *p* < 0.001
