# Supplementary material for: Healthcare workers’ experiences with COVID-19-related prevention and control measures in Tanzania
Source: PLOS Glob Public Health. 2023 Dec 5;3(12):e0002678. doi: 10.1371/journal.pgph.0002678 (PMC10697532; doi:10.1371/journal.pgph.0002678)
Supplement: Attachment — Submitted filename: Reviewer comments 2023 25 Sept final version.docx [file pgph.0002678.s003.docx]

September 25, 2023

Dear Dr. Hugh Cowley,

We are pleased to resubmit our manuscript titled "Healthcare Workers' Experiences with COVID-19-Related Prevention and Control Measures in Tanzania" to PLoS Global Public Health. We extend our heartfelt gratitude to you, the dedicated members of your editorial team, and the expert reviewers who invested their valuable time in evaluating our work and providing insightful feedback. We have incorporated these suggestions, leading us to gain invaluable insights and enhance the quality of our manuscript significantly.

In preparation for resubmission, we have addressed two distinct sets of feedback: the first originating from the journal's specific requirements and the second emanating from external peer reviewers. The feedback stemming from the journal's guidelines necessitated the inclusion of a copy of PLOS' questionnaire, additional elaboration on participant consent procedures, and the provision of the figure file. These modifications and our responses are comprehensively presented in Part A, which can be found in the attached table at the conclusion of this letter.

Part B of the table encompasses our responses to the constructive feedback provided by Reviewers #1 and #2. We have taken their reflections into consideration while revising our manuscript, aiming to enhance its overall quality and clarity.

We look forward to receive your feedback on this revised manuscript and would like to express our sincere appreciation once more for your invaluable guidance in the continual improvement of our paper.

Sincerely,

Kassimu Tani

**Journal requirement/Editor Feedback, Reviewers Comments and Authors’ Response**

|  | **Comments** | **Responses** |
| --- | --- | --- |
| **A** | **Journal Requirements:** |  |
| 1 | Please include a complete copy of PLOS’ questionnaire on inclusivity in global research in your revised manuscript. Our policy for research in this area aims to improve transparency in the reporting of research performed outside of researchers’ own country or community. The policy applies to researchers who have travelled to a different country to conduct research, research with Indigenous populations or their lands, and research on cultural artefacts. The questionnaire can also be requested at the journal’s discretion for any other submissions, even if these conditions are not met. Please find more information on the policy and a link to download a blank copy of the questionnaire here: <https://journals.plos.org/globalpublichealth/s/best-practices-in-research-reporting>. Please upload a completed version of your questionnaire as Supporting Information when you resubmit your manuscript. | Thank you: The questionnaire has been included in the submission as supporting information (S1 file) and the “Inclusivity in global research” section has been included in methods section. |
|  | Please provide additional details regarding participant consent. In the ethics statement in the Methods and online submission information, please ensure that you have specified (1) whether consent was informed and (2) what type you obtained (for instance, written or verbal, and if verbal, how it was documented and witnessed). If your study included minors, state whether you obtained consent from parents or guardians. If the need for consent was waived by the ethics committee, please include this information.  If you are reporting a retrospective study of medical records or archived samples, please ensure that you have discussed whether all data were fully anonymized before you accessed them and/or whether the IRB or ethics committee waived the requirement for informed consent. If patients provided informed written consent to have data from their medical records used in research, please include this information." | Apologies for the lack of detail:  Written informed consent was obtained prior to the participation in the survey. As the survey was online, participants were directed to the survey link only after ticking the boxes requiring to say they have read the information sheet, they have understood and ready to continue with survey. To address this comment, the text was revised and now reads:  “Written informed consent was obtained prior to the start of the survey, and respondents were required to confirm their understanding and intent to proceed by ticking three boxes, providing written confirmation, and signing the smartphone used to complete the survey in order to proceed to the survey. The survey was end-to-end encrypted to ensure respondents' privacy, and data was sent to a secure server at the Ifakara Health Institute. |
| 3 | Please provide separate figure files in .tif or .eps format.  For more information about figure files please see our guidelines:  <https://journals.plos.org/globalpublichealth/s/figures>  <https://journals.plos.org/globalpublichealth/s/figures#loc-file-requirements> | The figures have now been uploaded as separate file in .tif format. |
|  |  |  |
| **B** | **Reviewers' comments:** |  |
|  | **Reviewer #1:** |  |
| 1 | This manuscript presents an important and timely research topic that is the first of kind in not only Tanzania but in many sub-regions on the African continent. Healthcare workers (HCWs) experiences of implementing national and regional COVID-19 guidelines was certainly daunting in many parts of the world as research has shown but to capture and reveal what healthcare workers in sub-Saharan African HCWs experienced is novel by the authors and showcases the magnitude of what other HCWs encountered throughout the COVID-19 pandemic. | Thank you for your kind feedback. |
| 2 | I have read the manuscript several times and have concluded that the authors conducted the study in a novel and appropriate manner adhering to research ethics guidelines and step-wise procedure. The methodology including the study design, settings, sampling, and data collection were thoroughly described in clear language. The probability sampling used to select the districts was appropriate even though the individual HCW sampling was not very clear. My concern in the data collection is whether the Enkato and ODK were adopted without modification or they were modified. Again, did the researchers pilot test the instruments prior to collecting data? | Thanks for raising this issue. We have amended the method section to more clearly explain the HCW sampling.  We used Enketo and ODK as explained in the text.  We did pilot test the questionnaire before starting data collection.  The questionnaire was designed in XLS format and then uploaded to ODK and Enketo. All data collected was automatically sent to the Ifakara Health Institute server. |
| 3 | The statistical analysis in my opinion was robust and clearly presents the findings of the study that I believe many readers will understand and appreciate but I also believe that the write up of the results could be presented in a more step-by-step manners for most readers. The tables are presented very clearly nonetheless. | Thank you for this comment. We have carefully edited the manuscript and done our best to make the results section clearer and easier for readers to follow. |
|  | The discussion is well presented and reflects what the relationships of the findings are to existing literature. In addition, I would recommend adding examples of what other studies specifically reported rather than merely indicating that the findings are consistent or similar to what others have found. | Thanks for this suggestion. We have added a few examples of other studies in the discussion section. |
|  | Overall, this is great and timely study that sets the pace for other researchers in others sub-Saharan countries to replicate so that governments and healthcare authorities get to understand the treatment and guideline dynamics and the safety and well-being of HCWs in times of pandemics. This will help plan better and provide appropriate interventions for all cadres of HCWs. | Thank you for your kind feedback. |
|  |  |  |
|  | **Reviewer #2:** |  |
|  | Thank you for the opportunity to review this manuscript. The study aims to investigate the HCW:s experience of COVID-19 related prevention and control measures implemented in Tanzania.  The subject is interesting and there is a great need for systematically investigate and learn from the COVID-19 pandemic, especially in low-income countries. However, I have some major concerns, mainly regarding methods and the interpretation of the findings in this study, that needs some further efforts to entangle. | Thank you for your kind words. |
|  | **Major concerns** |  |
| 1 | There is a discrepancy between the title, aim and conclusions in the manuscript which needs to be addressed. The title includes the terms “compliance” and “burnout” which has not been assessed in the study and should be removed from the title. | We agree that the title went a bit beyond the content of the manuscript and have revised it to “Healthcare workers' experiences with COVID-19-related prevention and control measures in Tanzania” |
| 2 | In this study, a project specific COVID-19 questionnaire was used (as in most similar studies). How was this questionnaire developed, tested a validated? The ability of the questionnaire to accurately measure the correct aspects within the study will highly affect the possibility interpret the findings correctly. The authors need to add information on how the questionnaire was developed, tested and validated. | Thank you for raising this issue. To address this comment we have further described how the questionnaire was developed and tested.  In the methods section we added the following text:  “We developed the questionnaire using an iterative approach. We first reviewed the relevant literature and international and national guidelines on COVID-19 management. We then sought input from senior medical researchers with experience of health services in Tanzania.  After this valuable consultation, we submitted the questionnaire to the Tanzanian Ethics Committee, which provided insightful feedback. We incorporated their recommendations to refine the questionnaire. The revised version was then distributed to a select group of healthcare providers via their WhatsApp numbers. We carefully considered and responded to the feedback we received, making the necessary adjustments to ensure the effectiveness of the questionnaire. |
| 3 | In the data analysis, descriptive data has been calculated, differences have been investigated using chi-square tests and multivariate regression analysis have been conducted. The descriptive analyses have been described but the multivariate analyses need to be described more in detail to facilitate the reading and interpretation of the findings.  Firstly, the rationale for performing these analyses (“to examine the association between HCW and health facility characteristics and the composite implementation scores, challenges faced, and perception Likert scale scores”) is quite vague and could be explained more in detail.  Secondly, a long list of independent variables is presented, where all these included in the same model or where they used individually? Were the analyses adjusted for any of the background variables and why?  Thirdly, overall composite scores and separate composite scores were calculated and used in the analyses. The authors need to justify this and provide detailed information on whether these scores accurately reflect general precaution, changes in protocols to control etc. and how these scores may be interpreted (or refrain from using composite scores) | Thank you for the comment. To address this comment we have amended the methods section providing further explanation on the multivariate analysis.  1) We have revised the text to enhance its clarity.  The amended text from line 152 to line 169 (version with track changes) reads:  “Multivariate regressions were conducted to explore the relationship between the implementation of guidelines, challenges encountered by HCWs, and their perceptions of the implementation of COVID-19 control and treatment policies, taking into account specific characteristics of both HCWs and health facilities. The dependent variables under consideration included scores for guidelines implementation, challenges faced by HCWs, and HCWs' perceptions on a Likert scale.”  Independent variables included HCW age (grouped into three categories to reflect differential risk of severe COVID-19 infection or death for young adults, middle age adults, and older adults: 18-29, 30-49 and 50+) (36), gender, cadre, health facility level, ownership, rural/urban location, whether facility was designated as an official COVID-19 treatment center, availability of COVID-19 testing, whether facility had treated suspected or confirmed COVID-19 cases, institutions issuing COVID-19 guidelines (MOH, regional, district or local authorities), and whether HCWs were aware of COVID-19 guidelines. We specified three models for each outcome indicator. The first model used was a linear regression for implementation scores. It aimed to measure the relationship between existing predictors of implementation of guidelines for prevention and treatment of COVID-19 within health facilities. The second model was a logistic regression for the Likert scale scores of HCW perception of protection and well-being. The third model was a logistic regression intended to measure the predictors of reported challenges faced by HCWs. Standard errors were clustered at the district level, and all analyses were conducted using Stata IC 16. P value of <0.05 was considered statistically significant.”  2) Yes it is correct, all the listed independent variables presented, were included in all regression models. The variables included are listed in Table 3.  3) We have added further information on how the composite scores were computed. We believe that the composite scores are useful for the analysis.  At page 6 – from line 138 to line 146, we provided an explanation of what we did to generate the composite scores  “We employed a five-point Likert scale encompassing 13 perception-based questions regarding the implementation of COVID-19 control and treatment guidelines. This scale ranged from 1, indicating 'strongly disagree,' to 5, signifying 'strongly agree.' To maintain uniformity and consistency, we applied reverse coding to questions with negative wording, ensuring that a rating of 5 consistently represented a positive attitude.  In our data analysis, we utilized an overarching perception scale that combined all Likert-style scores. Furthermore, we generated two distinct component scores. The first encapsulated the responses to 7 questions pertaining to the health and well-being of HCWs, while the second encompassed the 6 questions related to COVID-19 treatment and guidelines.” |
| 4 | The interpretation of the findings needs to be revised in order to make sure that conclusions are drawn from the data at hand.  Firstly, the HCW:s situation during normal operations before the COVID-19 pandemic need to be acknowledge and how this will affect the findings in this study. I.e. the authors need to separate the effect from the pandemic from normal operations. For instance (but not limited to), on line 296-297, the authors state that “majority of HCWs reported fear of violence, while fewer HCWs reported increased stress and concern about putting their families at risk because of their work”. Looking at the questionnaire, 2 out of three questions supporting these findings are not specifically connected to the pandemic. How common is it for HCW:s to experiencing violence under normal operations? How should these results be interpreted in light of the aim of this study?  Secondly, on line 213-216 the authors state that there was a considerable variation in guideline implementation between different facilities. Is this difference caused by differences in implementation (as suggested by the author) or could it be caused by differences in the healthcare facilities (see table 1)? Is it also plausible to assume that all facilities would have 100% implementation on all guidelines regardless of the nature of their operations? All facilities might have the possibilities to use masks, but could all facilities postpone all routine visits, or will certain healthcare facilities have patient groups with for instance chronic diseases which requires medical surveillance regardless of the pandemic? How should these differences be interpreted and how will it affect the authors findings/conclusions? | Thank you for the pointing this out and here is the clarification.  All the questions included in the questionnaire were related to the COVID-19 period. However, we agree with the reviewer that in principle it would have been great to have a baseline pre COVID-19 to compare with. Unfortunately, it was not possible.  To address this comment we have amended the results and discussion section. The text now on line 316-317 result section reads “Few HCWs reported fear of violence, while the majority of them reported increased stress and concern about putting their families at risk because of their work (Figure 2).”  In the discussion section, the text on line 369-375 now reads,  HCWs in Tanzania reported heightened stress levels attributed to their work with infectious diseases. This stress stems from concerns about contracting infections themselves or transmitting them to others, largely due to the inadequacies in pandemic preparedness within the healthcare system. These findings align with prior studies that have documented deficiencies in health system preparedness, interruptions in healthcare services, and the psychological distress experienced by HCWs (40-42). Mitigating these fears and addressing other related concerns should be regarded as a pivotal component of an effective pandemic response (31, 43).  Second: The variation is mainly due to differences in implementation. In reviewing the guidelines distributed, it was clear that there was no differentiation based on the level of the facility. This formed the basis for our assumption that all facilities were expected to implement these guidelines, regardless of the type of services they provided. |
|  | **Minor concerns** |  |
| 1 | The background contains adequate information about the management of the pandemic in Tanzania but lacks information on the need of covid-care or the covid-19 disease burden during the time of the study which needs to be added in order to be able to compare the findings with other studies now and in the future. | Thank you for the comment:  Unfortunately, Tanzania did not disclosed detailed information on the number of covid-19 cases as explained in the Introduction. The study was conducted from December 2021 to March 2022. |
| 2 | Make sure that Table 1 contains all information necessary to be interpreted such as explaining that n (%) are presented in the table. Also, revise the table to make sure that the number of decimals is consistent throughout the table and whether to write 1000 or 1,000 for values above 999. P-values <0.000 should also be changes to <0.001 | Thank you for this comment, We have revised Table 1 accordingly. |
| 3 | The abbreviation MOH (line 146) has not been explained in the text | Thank you for spotting this inaccuracy. We have now spelt our Ministry of Health in line 98. |
| 4 | On line 220 and onwards, results from the regression analyses are presented. It would benefit the reader to be clear about which factors that are analysed instead of writing “have a larger effect size compared to the reference group” etc. | Thank you for the comment: we have amended the results section throughout to adding the factors analysed. |
| 5 | Line 319-322 seems to contain a discussion of the result rather that presenting findings from the study. The authors should consider moving this part to the discussion section. | Thank you for the comment. This has shifted to the discussion section. |
