# Supplementary material for: Healthcare workers’ experiences with COVID-19-related prevention and control measures in Tanzania
Source: PLOS Glob Public Health. 2023 Dec 5;3(12):e0002678. doi: 10.1371/journal.pgph.0002678 (PMC10697532; doi:10.1371/journal.pgph.0002678)
Supplement: Attachment — Submitted filename: Reviewer comment Novermber 2023.docx [file pgph.0002678.s004.docx]

November 3, 2023

Dear Dr. Landenmark,

We are pleased to resubmit our manuscript titled "Healthcare Workers' Experiences with COVID-19-Related Prevention and Control Measures in Tanzania" to PLoS Global Public Health. We extend our heartfelt gratitude to you, the dedicated members of your editorial team, and the expert reviewers who invested their valuable time in evaluating our work and providing insightful feedback. We have incorporated these suggestions, leading us to enhance the quality of our manuscript significantly.

In preparation for resubmission, we have addressed two distinct sets of feedback: the first originating from the journal's specific requirements and the second emanating from external peer reviewer. The feedback stemming from the journal's guidelines necessitated the reference list to ensure that it is complete and correct. The references modifications and our responses are comprehensively presented in Part A which can be found in the attached table at the conclusion of this letter.

Part B of the table encompasses our responses to the constructive feedback provided by Reviewers #1 and #2. We have taken their reflections into consideration while revising our manuscript, aiming to enhance its overall quality and clarity.

We look forward to receiving your feedback on this revised manuscript and would like to express our sincere appreciation once more for your guidance and support.

Sincerely,

Kassimu Tani

|  | **Comments** | **Responses** |
| --- | --- | --- |
| **A** | **Journal Requirements:** |  |
| 1 | Please review your reference list to ensure that it is complete and correct. If you have cited papers that have been retracted, please include the rationale for doing so in the manuscript text, or remove these references and replace them with relevant current references. Any changes to the reference list should be mentioned in the rebuttal letter that accompanies your revised manuscript. If you need to cite a retracted article, indicate the article’s retracted status in the References list and also include a citation and full reference for the retraction notice. | All references have been re-checked for their completeness. |
|  |  |  |
| **B** | **Reviewers' comments:** |  |
|  | Reviewer #1: |  |
| 1 | All comments that I made in my last review have been adequately address. The only new thing I have observed that the authors should try and present the result on challenges in categories to make them more clearer to the reader. It appears that the reported challenges faced by the HCWs are lumped together. | Thank you for this suggestion. We have amended the text to provide a more structured overview of the challenges that are now grouped in categories.  The amended text from line 280 – 315 reads:  HCWs reported facing the following challenges:   - **Stress related to risks of infections:** 73.0% of HCWs experienced stress related to working with a infectious diseases, primarily at hospitals (75.9%) and referral hospitals (74.0%) (Figure 1 and Appendix Table C). Among these, 64.1% expressed concern about their own infection or the risk of transmitting the disease with the majority at hospitals (69.1%) and referral hospitals (67.5%). - **Emotional distress from limited treatment options:** 32.1% of respondents reported emotional distress due to limited treatment options for deteriorating patients, with the highest incidence recorded at referral hospitals (40.8%). - **Emotional distress from high mortality:** Additionally, 22.6% reported emotional distress due to the increased mortality rate, primarily at hospitals and referral hospitals (26.6%). - **Exhaustion from protective equipment:** Wearing additional protective equipment led to exhaustion in 22.3% of HCWs, most notably at hospitals (25.1%) and clinics (23.9%). - **Fatigue from increased workload:** Furthermore, 15.2% of HCWs experienced fatigue due to an increased workload, primarily at referral hospitals (20.6%). - **Threats and violence:** A concerning 15.8% of HCWs reported experiencing threats or violence, with the highest incidents reported at referral hospitals (21.2%) (Figure 1 and Appendix Table C).   Variations among HCWs:   - **Gender Differences:** Female HCWs had lower odds of reporting stress from working with an infectious disease (0.74 CI: 0.68, 0.81) and exhaustion from wearing protective equipment (0.79 CI: 0.69, 0.90), but higher odds of reporting worry about the uncertainty of becoming infected or infecting others (1.25 CI: 1.10, 1.42), as well as emotional distress from powerlessness when patients deteriorate (1.34 CI: 1.12, 1.49) or high mortality (1.31 CI: 1.17, 1.46) (Table 4). - **Occupation differences:** Doctors reported higher odds of experiencing most of the challenges we specifically asked about, including threats or violence against HCWs (1.71 CI: 1.37, 2.13), exhaustion from wearing protective gear (2.02 CI: 1.58, 2.59), fatigue from increased workload (1.32 CI: 1.09, 1.59), fear of infection (1.24 CI: 1.06, 1.46), and emotional distress from high mortality (1.33 CI: 1.13, 1.56). - **Urban vs. rural differences:** HCWs in urban facilities also had higher odds of many of the challenges listed, including violence against HCWs (1.27 CI: 1.06, 1.51), exhaustion from wearing protective gear (1.27 CI: 1.08, 1.49), fatigue from increased workload (1.33 CI: 1.10, 1.61), emotional distress from feeling powerless when patients deteriorate (1.15 CI: 1.01, 1.30), and emotional distress from high mortality (1.31 CI: 1.13, 1.50). - **Health facility level:** There were minimal differences in the challenges faced by HCWs across health facility levels, except for the fear of becoming infected or infecting others, where the odds of reporting this challenge were higher in health centers (1.34 CI: 1.17, 1.53), hospitals (1.57 CI: 1.32, 1.87), and referral hospitals (1.50 CI: 1.10, 2.05) compared with dispensaries (Table 4). |
| 2 | Lines 309-313 talk about HCWs perception of the health system response where the authors used terms such as agreements and strong agreement without specifying exactly what HCWs agreed on. Also, the use of MOST without indicating the necessary proportions or percentages. Please, clarify these. | Thank you for the pointing this out and here is the clarification. To address this comment we amended the mentioned paragraph (line 330 -344 in the new version) and the text now reads:  “In terms of HCWs' views on the health system's response to COVID-19, there were high levels of agreement and strong agreement on questions related to the clarity and sources of information on COVID-19 guidelines, as well as the duty of HCWs to protect patients, even at the risk of their own and their families' health. In addition, 77% of HCWs believe that facility management cares about their well-being (Figure 2).  However, some questions received more mixed responses, with HCWs showing varying degrees of agreement and disagreement. Most HCWs felt that the policies in place to protect them were adequate (67%) and that they felt safe at work (58%). However, 53% reported a lack of personal protective equipment (PPE). Few HCWs reported fear of violence (23%), while 53% of them reported increased stress and concern about putting their families at risk because of their work (Figure 2).  The survey results suggest that HCWs received clear guidance (89%) and were strongly committed to the well-being of patients (69%). However, 47% of HCWs felt that health facilities were ill-prepared to handle COVID-19 and 43% reported concerns about the lack of personal protective equipment (PPE). Although many HCWs feel supported by their facility management and are committed to protecting patients, (53%) reported that their concerns about COVID-19 treatment have not been adequately addressed (Figure 2).”  Also we have amended Figure 2, by labeling the right hand side of the graph (agree and strong agreed) and the left hand side the disagree and strong disagreement. |
| 3 | In all, I think the authors addressed the comments and strengthened the manuscripts quality and originality. | Thank you for your kind feedback. |
|  | **Reviewer #2:** |  |
|  | (No Response) | Thank you for your kind feedback. |
